# Serum neurofilament light chain as a predictive marker of neurologic outcome after cardiac arrest: a meta-analysis

**DOI:** 10.1186/s12872-023-03220-z

**Published:** 2023-04-15

**Authors:** Shu Li Wang, Nan Li, Shun Yi Feng, Yong Li

**Affiliations:** Emergency Deparment, Cangzhou Central Hospital, No.16 Xinhua Road, Yunhe Qu, Cangzhou City, 061000 China

**Keywords:** Cardiac arrest, Cardiopulmonary resuscitation, Brain injury, Neurofilament light

## Abstract

**Objective:**

Recently, an increasing number of studies have suggested using serum neurofilament light (NfL) chain to predict the neurologic outcome after cardiac arrest. However, the predictive ability of this approach remains inconclusive. Meta-analysis was performed on related studies to assess the ability of serum NfL to predict the neurologic outcome after cardiac arrest.

**Materials and methods:**

PubMed, ScienceDirect and Embase were systematically searched from the date of their inception until June 2022. Data were extracted to calculate the area under the receiver operating characteristic curve (AUC), the sensitivity, the specificity and the publication bias to evaluate the predictive power of serum NfL using Stata 14.0.

**Results:**

Nine studies were included in the present meta-analysis. Seven studies involving 1296 participants reported serum NfL 24 h post arrest for predicting the neurological outcome, and the AUC was 0.92 (77% sensitivity and 96% specificity). Seven studies involving 1020 participants reported serum NfL 48 h post arrest for predicting the neurological outcome, and the AUC was 0.94 (78% sensitivity and 98% specificity). Four studies involving 804 participants reported serum NfL 72 h post arrest for predicting the neurological outcome, and the AUC was 0.96 (90% sensitivity and 98% specificity). No significant publication bias was observed among the included studies.

**Conclusion:**

The present meta-analysis results support the potential use of serum NfL as an early biomarker of neurologic outcome, especially 72 h post arrest.

**Supplementary Information:**

The online version contains supplementary material available at 10.1186/s12872-023-03220-z.

## Introduction

Cardiac arrest, a fatal and an uncommon event caused by acute myocardial infarction [1, 2], pesticide poisoning, [3] trauma, [4] etc., has an estimated average global incidence of approximately 100 cardiac arrests per 100,000 persons among adults [5, 6]. The rate of survival to discharge is 10% in patients with out-of-hospital cardiac arrest worldwide, and 22% in patients with in-hospital cardiac arrest [7–9]. Despite the progress in advanced life support, only less than 10% of survivors have good neurological outcomes when discharged from hospitals [10, 11].

Neurofilament light chain (NfL), an axonal structural protein, is an established biomarker for axonal damage [12–14]. Some studies have assessed the association between serum NfL and neurologic outcomes after cardiac arrest, but the results are inconclusive [15–23]. Therefore, this systematic review and meta-analysis aim to investigate the predictive value of serum NfL for neurologic outcomes after cardiac arrest.

## Materials and methods

This study was conducted and reported according to the Preferred Reporting Items for Systematic Reviews and Meta-Analyses statement. This study does not require ethical approval because the meta-analysis is based on published research, and the original data are anonymous.

### Literature search

PubMed, ScienceDirect and Embase were systematically searched from the date of their inception until June 2022. The peer-reviewed search strategy includes the combination of subject headings and free text with terms, such as ‘neurofilament light chain [Title/abstract]’ and ‘cardiac arrest [Title/abstract]’. In addition, the references of the included investigations were reviewed.

### Inclusion and exclusion criteria

The eligibility of individual studies was evaluated by two investigators, who independently used the predefined exclusion/inclusion criteria. Studies were selected according to the following criteria: (1) study type: prospective or retrospective studies; (2) patients: the participants experienced cardiac arrest; (3) intervention measures: use of serum NfL to predict the neurologic outcome after cardiac arrest; (4) sufficient data are provided for neurologic outcomes, and detailed information are available. The exclusion criteria were as follows: (a) insufficient data for outcome evaluation even after contacting the authors; (b) no reported sensitivity and specificity or true positive, false positive, true negative and false negative cases; (c) abstracts, editorials, narrative reviews, case reports and letters to the editor; and (d) sample size < 20.

### Data extraction

Two reviewers independently abstracted the following information from the included studies: first author, publication year, sex, study country, study design, proportion of poor neurologic outcome, arrest location, and targeted temperature management. Authors were contacted when further clarification was required. Differences were resolved by reviewing the included studies until a consensus was reached.

### Quality assessment

The quality of each included study was assessed and scored by two independent reviewers using the Quality Assessment of Diagnostic Accuracy Studies 2 questionnaire. The following domains were used to evaluate each study: the selection of the patient, the index test, the reference standard, and the flow and timing. Differences in the assessment were discussed and resolved with consensus among investigators.

### Statistical analysis

The Stata version 14.0 software (StataCorp LLC, College Station, Texas, USA) was used for the statistical analysis of all the data. For dichotomous outcomes, we calculated the odds ratios (ORs) with their corresponding 95% confidence intervals (CIs). *I*^2^ statistics were used to assess the presence of heterogeneity. If the heterogeneity test had a *P*-value < 0.1, then a random effects model was used; otherwise, the fixed effects model was used. Meta-regression analysis was performed to identify the sources of heterogeneity. Sensitivity analysis was conducted by pooling the estimates to determine the robustness of our conclusions. The publication bias among the studies was assessed by using Deek’s funnel plot asymmetry test. The level of significance was set at *P* < 0.05.

## Results

### Literature search

**Fig. 1:**
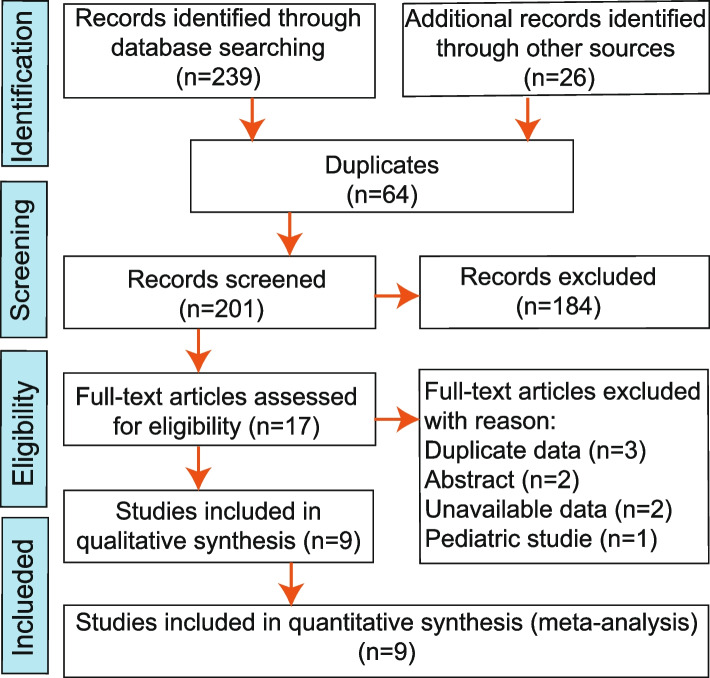
Flow chart for the selection of studies

The study selection process is illustrated in Fig. 1. Our initial search of the electronic databases yielded 265 candidate studies. Following de-duplication, 201 titles and abstracts were screened, and 16 studies were identified. Seven studies were excluded after a full-paper analysis. Finally, nine studies [15–23] were included for meta-analysis.

### Characteristics and quality assessment of the included studies

Table 1 shows the characteristics and quality assessment of the nine included studies. Most of the included studies were conducted in Europe (78%). Approximately 67% (6/9) of the studies had a retrospective design, and 11% (1/9) selected out-of-hospital cardiac arrest as the research object.

**Table 1:** Characteristics of the nine studies included

	**Age**	**Male**	**Study country**	**Study design**	**Sample size**	**Poor neurologic outcome**	**Cut-off value (pg/ml)**			**OHCA**	**TTM**
							**24 h**	**48 h**	**72 h**		
Adler 2021 [15]	64 ± 12	83%	Germany	Retrospective	53	43.4%	241.7	508.6	NA	100%	100%
Huesgen 2021 [16]	60	68%	America	Prospective	22	54.5%	2787	5237	16,859	100%	72.7%
Hunziker 2021 [17]	63 ± 15	71%	Switzerland	Prospective	164	59.8%	50	NA	NA	100%	66.5%
Moseby–Knappe 2021 [18]	65	81%	Sweden	Retrospective	692	56.5%	55	55	55	100%	50.1%
Pouplet 2022 [19]	62	82%	France	Retrospective	49	46.9%	NA	500	NA	91.8%	NA
Rana 2013 [20]	63	74%	Germany	Prospective	61	41.9%	323	405	309	100%	NA
Wihersaari 2021 [21]	62	82%	Finland and Denmark	Retrospective	112	34.8%	127	263	344	100%	100%
Wihersaari 2022 [22]	63	84%	Finland	Retrospective	197	48.4%	232	NA	NA	100%	77.4%
Wurm 2021 [23]	56	76%	Austria	Retrospective	70	70.0%	NA	NA	NA	100%	100%

Age is given as median or mean ± standard deviation

*OHCA* Out-of-hospital cardiac arrest, *TTM*  Targeted temperature management

### Quality assessment

The quality plots were completed using Review Manager 5.3.0 and are shown in supplementary Fig. 1. A high risk of bias mainly existed when the threshold was pre-specified and the patients were consecutive enrolled.

### Comparison of serum NfL levels across groups and time points

**Fig. 2:**
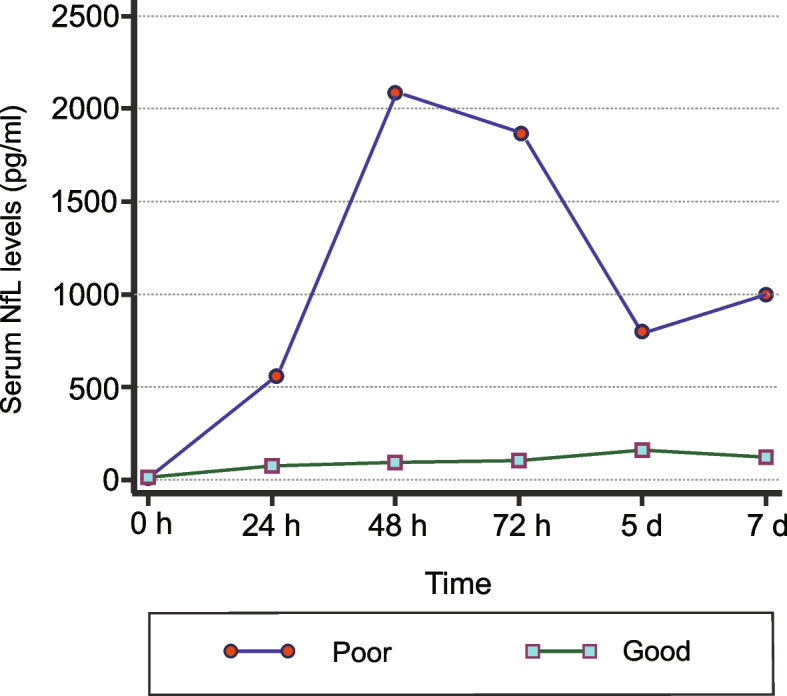
Comparison of serum NfL levels across groups and time points. *NfL* Neurofilament light

Three studies compared the serum NfL levels across groups and time points [16, 20, 21]. The serum NfL levels increased immediately in patients with a poor neurological outcome at hospital admission, reached peak values from 48 to 72 h, and then gradually decreased and maintained a steady level. On the contrary, the serum NfL level was at a relatively constant low level in patients with a neurological outcome (Fig. 2).

### Serum NfL at 24 h post arrest for predicting poor neurological outcome

**Fig. 3:**
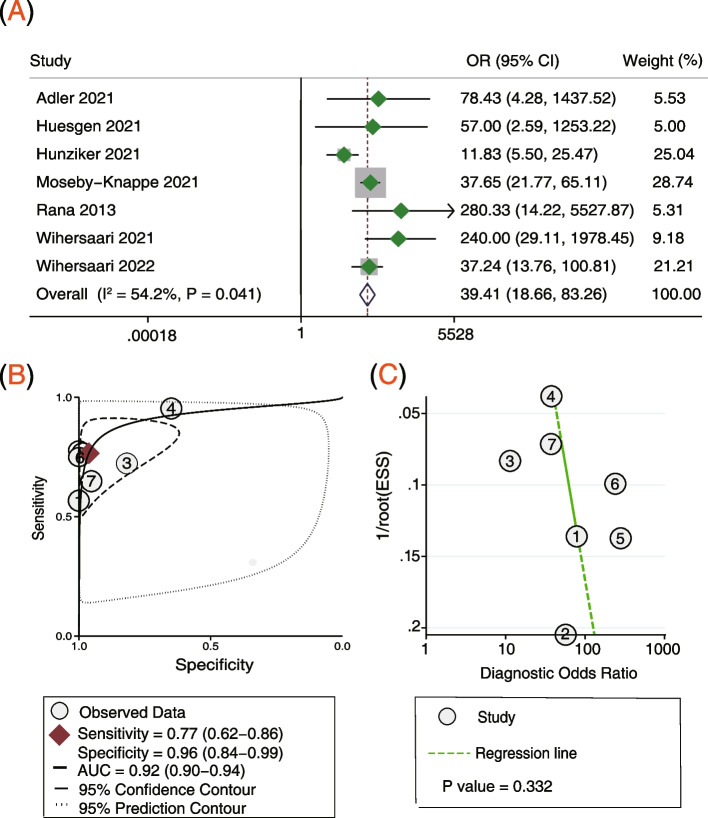
Serum NfL levels 24 h post arrest for predicting poor neurological outcome. **A** Forest plot of the ORs for NfL and poor neurological outcome, **B** summary receiver operating characteristics curve of NfL for predicting poor neurological outcome and **C** funnel plot for detecting publication bias. *NfL* Neurofilament light

Seven studies [15–18, 20–22] involving 1296 participants reported the serum NfL 24 h post arrest for predicting the neurological outcome. Pooled analysis showed that the serum NfL 24 h post arrest was correlated with poor neurological outcome (OR = 39.41, 95% CI: 18.66–83.26, *P* < 0.001; *I*^2^ = 54.2%, *P* = 0.041; Fig. 3A). The AUC of the serum NfL 24 h post arrest was 0.92, with a sensitivity of 77% and a specificity of 96% (Fig. 3B). Deek’s funnel plot asymmetry test suggests no publication bias (*P* = 0.332; Fig. 3C).

### Serum NfL at 48 h post arrest for predicting poor neurological outcome

**Fig. 4:**
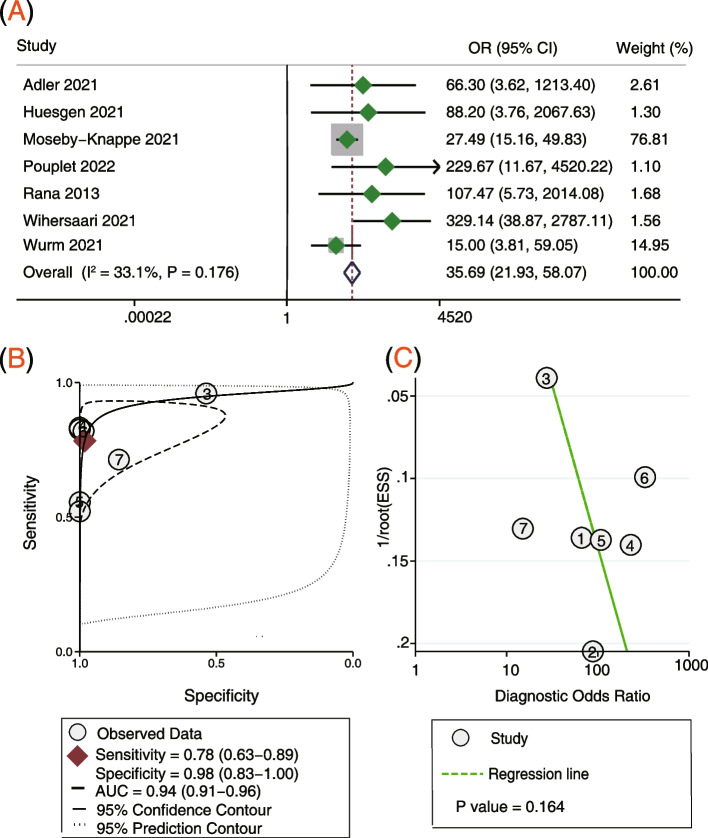
Serum NfL levels 48 h post arrest for predicting poor neurological outcome. **A** Forest plot of ORs for NfL and poor neurological outcome, **B** summary receiver operating characteristics curve of NfL for predicting poor neurological outcome, and **C** funnel plot for detecting publication bias. *NfL* Neurofilament light

Seven studies [15, 16, 18–21, 23] involving 1020 participants reported the serum NfL 48 h post arrest for predicting the neurological outcome. Pooled analysis showed that the serum NfL 48 h post arrest was correlated with poor neurological outcome (OR = 35.69, 95% CI: 21.93–58.07, *P* < 0.001; *I*^2^ = 33.1%, *P* = 0.176; Fig. 4A). The sensitivity was 78%, the specificity was 98%, and the AUC was 0.94 (Fig. 4B). Deek’s funnel plot asymmetry test suggests no publication bias (*P* = 0.164; Fig. 4C).

### Serum NfL at 72 h post arrest for predicting poor neurological outcome

**Fig. 5:**
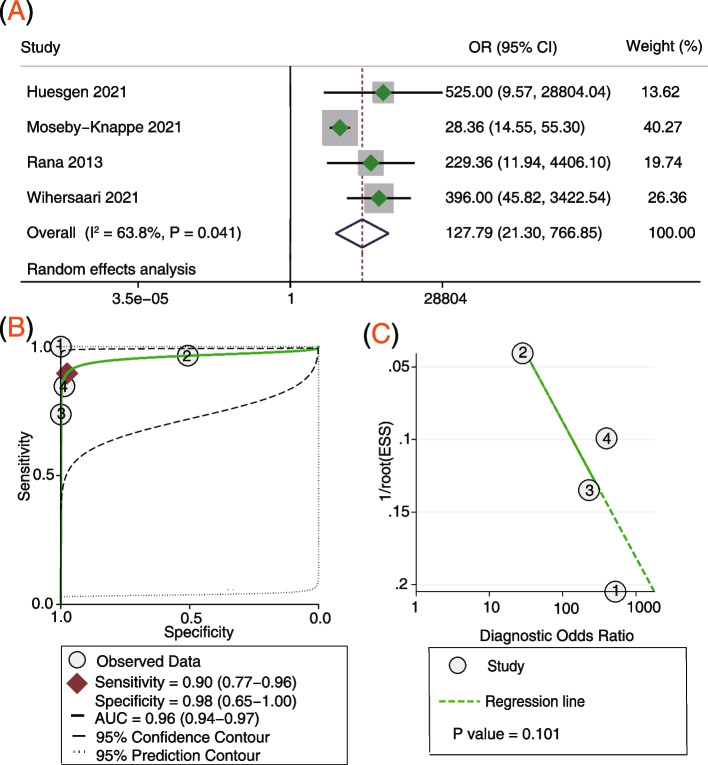
Serum NfL levels 72 h post arrest for predicting poor neurological outcome. **A** Forest plot of the ORs for NfL and poor neurological outcome, **B** Summary receiver operating characteristics curve of NfL for predicting poor neurological outcome and **C** funnel plot for detecting publication bias. *NfL* Neurofilament light

Four studies [16, 18, 20, 21] involving 804 participants reported the serum NfL 72 h post arrest for predicting the neurological outcome. Pooled analysis showed that the serum NfL 72 h post arrest was correlated with poor neurological outcome (OR = 127.79, 95% CI: 21.30–766.85, *P* < 0.001; *I*^2^ = 63.8%, *P* = 0.041; Fig. 5A). The sensitivity was 90%, the specificity was 98%, and the AUC was 0.96 (Fig. 5B). Deek’s funnel plot asymmetry test suggests a publication bias (*P* = 0.101; Fig. 5C).

### Meta-regression analysis and sensitivity analysis

The results of the meta-regression analysis show that age, gender, sample size, witnessed cardiac arrest, and shockable rhythm were not the source of heterogeneity for the serum NfL 24 and 48 h post arrest for predicting poor neurological outcome (Supplementary Tables 1 and 2). Given that the number of studies for the serum NfL 72 h post arrest for predicting the poor neurological outcome is rather small, meta-regression analysis cannot be performed. The robustness of the results was assessed using leave-out-one sensitivity analysis, which showed some influence of the individual studies on the pooled ORs (Supplementary Fig. 2).

## Discussion

The current biomarkers of neurologic outcomes after cardiac arrest are mainly based on serum S100 and neuron-specific enolase, but their prediction has been proven insensitive, especially for early detection. Developing a reliable diagnostic and prognostic biomarker in cardiac arrest management is urgently required. Therefore, the present meta-analysis emphasizes the importance of novel biomarkers for the early prediction of neurologic outcomes after cardiac arrest. The current evidence demonstrates that that serum NfL level was independently associated with neurologic outcomes and might be valuable biomarkers for the prognosis, especially 72 h post arrest.

Three studies [16, 20, 21] described the dynamic change in the serum NfL levels and the corresponding predictive performance at different time points. Wihersaari et al. [21] compared NfL data at four different time points (ICU admission and 24, 48 and 72 h after cardiac arrest) and indicated that the serum NfL 48 h after cardiac arrest exhibited the highest sensitivity (85%) when the specificity was set to 100%. In another study [20], the serum NfL at the 7th day had the highest sensitivity (94%) when the specificity was set to 100% after assessing the serum NfL data at five different time points (24 h, 48 h, 72 h, 5 days and 7 days after cardiac arrest). On the basis of the current evidence, we cannot determine the best time points for monitoring the serum NfL. However, the serum NfL, at most time points within 7 days, has excellent predictive performance, with > 80% sensitivity and near 100% specificity.

Notably, only three studies examined the predictive performance of the serum NfL 72 h post arrest for predicting the neurologic outcomes [24]. Although the overall effect size was not significantly influenced in the sensitivity analysis, between-study heterogeneity was found among the three studies. Thus, more related studies should explore the predictive value of the serum NfL 72 h post arrest for neurologic outcomes. In addition, only two studies [17, 25] on the role of the serum NfL for predicting the 30-day mortality were conducted. However, the results pooled based on the two studies are still encouraging, with 77% sensitivity and 83% specificity [17, 25].

In addition, the prognostic neurologic outcome was measured at different points in time, ranging from hospital discharge to 12 months after discharge. The neurologic outcome might change with time, and these patients who were initially identified as cerebral performance category 3 (‘poor’ neurologic outcome) would be converted to cerebral performance category 2 (‘good’ neurologic outcome) with time. Wurm et al. [23] reported that one in five of the cardiac arrest patients had a shift between favourable and unfavourable occurrence, which improved the predictive ability with the change in the AUC from 0.79 to 0.87.

Therapeutic hypothermia is widely used as an effective strategy to minimise brain damage in patients who achieve return of spontaneous circulation after cardiac arrest. Once the patient is started on therapeutic hypothermia, signs of neurological recovery are often delayed making the evaluation of the neurological function difficult. However, therapeutic hypothermia ICU care did not interfere with the prognostic accuracy of the serum NfL [21].

This meta-analysis serves as baseline information for further study and still has limitations. First, the results of our meta-analyses may be affected by the limited number of available studies and the small number of patients included. Second, the test methods for determining the serum NfL levels differed across the studies due to the lack of a uniform method of reporting the serum NfL level, probably causing test bias. Third, publication bias exists and may decrease the reliability of the results.

## Conclusion

Serum NfL may become an important tool for detecting the neurological outcome after cardiac arrest, potentially complementing existing methods of neurological prognosis for assessment, and providing early clinical predictions.

## Supplementary Information

**Additional file 1:** **Supplementary figure 1.**

**Additional file 2:** **Supplementary figure 2.**

**Additional file 3:** **Supplementary table 1.**

**Additional file 4:** **Supplementary table 2.**

## Data Availability

The datasets analysed during the current study are not publicly available due to privacy policy but are available from the corresponding author on reasonable request.

## Abbreviations

CPR: Cardiopulmonary resuscitation
IQR: Interquartile range
OHCA: Out-of-hospital cardiac arrest
ROC: Receiver operating characteristic curve
AUC: Area under the curve
NfL: Neurofilament light chain
SD: Standard deviation
TTM: Targeted temperature management
