# Supplementary material for: Serum neurofilament light chain as a predictive marker of neurologic outcome after cardiac arrest: a meta-analysis
Source: BMC Cardiovasc Disord. 2023 Apr 15;23:193. doi: 10.1186/s12872-023-03220-z (PMC10105388; doi:10.1186/s12872-023-03220-z)
Supplement: Supplementary file 3 — Additional file 3: Supplementary table 1. [file 12872_2023_3220_MOESM3_ESM.docx]

**Supplementary table 1 Meta-regression of heterogeneity for serum NfL levels 24 h post arrest**

| Heterogeneity factors | Coefficient | SE | Z | *P* value | 95% CI |
| --- | --- | --- | --- | --- | --- |
| Age | -0.183 | 1.22 | -0.15 | 0.905 | -15.67, 15.31 |
| Male | 1.78 | 1.32 | 1.35 | 0.406 | -15.02, 18.58 |
| Sample size | -1.14 | 0.97 | -1.18 | 0.448 | -13.48, 11.19 |
| Witnessed cardiac arrest | 1.79 | 1.21 | 1.48 | 0.379 | -13.62, 17.20 |
| Shockable rhythm | -0.67 | 1.36 | -0.49 | 0.708 | -17.94, 16.59 |

CI = confidence intervals; SE = standard error; NA = not available.
