# Supplementary material for: Serum neurofilament light chain as a predictive marker of neurologic outcome after cardiac arrest: a meta-analysis
Source: BMC Cardiovasc Disord. 2023 Apr 15;23:193. doi: 10.1186/s12872-023-03220-z (PMC10105388; doi:10.1186/s12872-023-03220-z)
Supplement: Supplementary file 4 — Additional file 4: Supplementary table 2. [file 12872_2023_3220_MOESM4_ESM.docx]

**Supplementary Table 2 Meta-regression of heterogeneity for serum NfL levels 48 h post arrest**

| Heterogeneity factors | Coefficient | SE | Z | *P* value | 95% CI |
| --- | --- | --- | --- | --- | --- |
| Age | -0.85 | 1.50 | -0.56 | 0.674 | -19.96, 18.27 |
| Male | 1.42 | 1.59 | 0.89 | 0.537 | -18.84, 21.67 |
| Sample size | -0.82 | 1.47 | -0.56 | 0.675 | -19.52, 17.87 |
| Witnessed cardiac arrest | 1.92 | 0.97 | 1.98 | 0.298 | -10.39, 14.22 |
| Shockable rhythm | -0.73 | 1.43 | -0.51 | 0.701 | -18.94, 17.49 |

CI = confidence intervals; SE = standard error; NA = not available.
